# Determinants of modern contraceptive use among sexually active men in Kenya

**DOI:** 10.1186/s12978-017-0316-3

**Published:** 2017-04-27

**Authors:** Rhoune Ochako, Marleen Temmerman, Mwende Mbondo, Ian Askew

**Affiliations:** 1 Faculty of Medicine and Health Sciences, Ghent University, Ghent, Belgium; 2 Population Council, P.O. Box 17643, 00100 Nairobi, Kenya; 3 International Centre for Reproductive Health, Ghent University, Ghent, Belgium; 4 Aga Khan University, Nairobi, Kenya; 5 CIVITRA Research and Consulting Company Ltd., Nairobi, Kenya; 6 World Health Organization, Geneva, Switzerland

**Keywords:** Modern contraceptive use, Men, Family planning, Kenya

## Abstract

**Background:**

Research in Kenya has focussed on family planning from women’s perspectives, with the aim of helping reduce the burden of unintended pregnancies. As such, the determinants of modern contraceptive use among sexually active women are well documented. However, the perspectives of men should be considered not only as women’s partners, but also as individuals with distinct reproductive histories and desires of their own. This study seeks to understand the determinants of modern contraceptive use among sexually active men, by exploring factors that are correlated with modern contraceptive use.

**Methods:**

The data source is the nationally representative 2014 Kenya Demographic and Health Survey (DHS) of men aged 15–54 years. The analysis is restricted to 9,514 men who reported being sexually active in the past 12 months prior to the survey, as they were likely to report either doing something or not to avoid or delay pregnancy. We use bivariate and multinomial logistic regression to assess factors that influence modern contraceptive use among sexually active men.

**Results:**

Findings from the bivariate and multinomial logistic regression indicate that region of residence, marital status, religion, wealth, interaction with a health care provider, fertility preference, number of sexual partners and access to media were all significantly associated with modern contraceptive use among sexually active men.

**Conclusion:**

Provider-client interaction as well as dissemination of information through mass media has the potential to increase knowledge and uptake of modern contraceptives. Similar efforts targeting segments of the population where contraceptive uptake is low are recommended.

## Plain English Summary

Men should be considered not only as women’s partners, but also as individuals with distinct reproductive histories and desires of their own. This study sought to understand the determinants of modern contraceptive use among sexually active men, by exploring factors that are associated with modern contraceptive use. Relative strength of these associations is explored in bivariate and multivariate models. Findings indicate that region of residence, place of residence, marital status, religion, wealth, interaction with a health care provider, fertility preference, number of sexual partners and having access to media were all significantly associated with modern contraceptive use among sexually active men. Provider-client interaction as well as dissemination of information through mass media has the potential to increase knowledge and uptake of modern contraceptives. Similar efforts targeting segments of the population where uptake is low is recommended.

## Background

The 2014 Kenya Demographic and Health Survey reports the contraceptive prevalence rate (CPR) for Kenya as 58% among married women, and 65% among sexually active unmarried women [1]. While CPR has steadily increased over the years, the same survey shows continued variances across the country based on age, region, level of education, among other determinants. For instance, married women from urban areas were found to have a CPR of 62% while those from rural areas were at 55%, and married women with secondary education or higher had a CPR of 65% while those with no education were at 18%. Similar findings have been documented in other sub-Saharan Africa countries including research from Ghana that showed increasing trends in contraceptive use when analysing data from 5 consecutive Ghana Demographic Health Surveys between 1988 and 2008, and residence as well as education being key determinants of contraceptive use [2]. Despite the steady improvement in CPR, it still falls short of the targets for the now defunct Millennium Development Goal set at 70% for Kenya. The FP2020 2015–2016 progress report shows that the unmet need for contraception for Kenya now stands at 20.1% [3], another indication that steady progress is being made towards achieving the Sustainable Development Goal 3, ‘ensure universal access to sexual and reproductive health-care services, including family planning, information and education, and the integration of reproductive health into national strategies and programmes by 2030’ [4].

Research in Kenya has focussed on family planning from women’s perspectives, with the aim to help reduce the burden of unintended pregnancies [5–7]. Despite this, men are important given their role in sex and reproduction. Additionally, population scientists have focused their study on fertility almost exclusively on the fertility behaviour of women while paying little attention to the role of men and the implication of their participation on fertility and population growth [5]. Several studies have highlighted the influence of men on reproductive decisions such as number of children and contraceptive use, noting that men’s influence may not necessarily reflect the reproductive decisions of their wives [6, 7]. A review of DHS data from Bangladesh, Dominican Republic and Zambia showed that calculated unmet need for wives differed from the calculated unmet need for husbands and couples [8], indicating that men also have their own fertility desires. Many family planning programmes also exclude the participation of men. Since men are the heads of households, they make decisions around the well-being of their households including decisions on family planning [9]. In recent years, efforts are underway to broaden men’s involvement in reproductive health and family planning. More specifically, measures are underway to improve gender relations and men’s understanding of their familial and social roles in family planning and sexual and reproductive health issues [10]. For a country like Kenya where population growth, HIV/AIDS and youth pregnancies are all serious issues for development, improving contraception uptake is an important priority for public health [11].

The role of others in influencing family planning use or non-use is well documented in Kenya [12–15]. Analysis of the 1994 Kenya Situation Survey found that women who had discussed family planning with both core and extended network members were 8 times as likely to be currently using modern contraceptives, and men who had done so were 3 times as likely as were those who had limited such discussions to their core network only [16]. In other parts of sub-Saharan Africa, research shows how social networks can strengthen positive messages among users; for example, in Cameroon a study found 55% of the sample reported how at least one network partner encouraged use of contraceptives [17, 18]. However, social networks can also propagate myths about family planning by exaggerating side effects and spreading rumours [13, 19, 20]. Findings from research by Ochako et. al, confirm that a major barrier to starting use of modern contraceptives among young women is myths and misconceptions, learned from others in their social network [6]. The decision for a woman to use contraception or not is primarily influenced by others, whose views and perceptions are often more important than an individual’s own [6, 12].

Gender and social norms play a key role in the decision to use or not to use contraception, with men playing a greater part in this decision [21, 22]. In particular, the views and perceptions of the husband/partner are key in determining contraceptive use [23–26]. A study in Kenya found that husbands had great decision making power and the ability to effect compliance or submission from their wives [27]. Husband’s approval of contraception is also crucial for successful family planning programmes. Studies have shown that family planning adoption is likely to be more effective for women when men are actively engaged by the programmes, through education or other targeted activities [28–30]. Although many researchers advocate for including men in family planning programs, data on men's knowledge and use of contraception remains scarce [24–26, 31–37]. Demographic studies on fertility and family planning, both quantitative and qualitative, large scale and small, have tended to focus on women alone [24, 38]. This is now changing slowly and a brief review of the published literature from Sub-Saharan Africa is set out below.

Vouking, Evina and Tadenfok analyzed data from several sub-Saharan countries on male involvement in family planning [39]. Their findings indicate that while male knowledge of family planning was almost universal, their involvement in the decision making process was not as straight-forward with a majority of men disagreeing that they should make decisions about selected family planning issues in the family. Further, female respondents were of the opinion that the selection of a contraceptive method was equally made by women or jointly, with male-dominated decisions falling last. Additional studies from sub-Saharan Africa add to the complexity of male involvement in family planning. For example, in Southwestern Nigeria, a study with men concluded that male involvement in family planning decision making was poor and their use of family planning services was low [40]. While in the same region, a different (more comprehensive) study found almost twice as many men as women consenting to the use of family planning with the male partner being highly motivated to obtain contraceptives, particularly in extramarital relationships [41].

Studies from Ethiopia have more consistent results in regards to men and family planning. In Tigray region, a cross-sectional survey found that over 90% of men supported and approved of using family planning; however, 36% of men did not know about male contraceptive methods [42]. Similarly, approval was 90% in Southern Ethiopia [23] and in Jimma Zone (93%), where only 4 out of 811 men ever used contraception [43]. In Northwest Ethiopia, a study with men found that only 8% of respondents were using or directly participating in the use of family planning services [44]. In Uganda, researchers used data from the 2011 DHS to identify factors that influenced modern contraceptive use among sexually active men. Findings indicated that discussion of family planning with a health worker, region, education, wealth index, number of surviving children and fertility preference were most significantly associated with modern contraceptive use among men [32]. One of the few published studies from Kenya on male involvement in family planning used focus group discussions to understand perceptions among low-income men in Western Kenya. This study found men’s knowledge of contraception inadequate, as their knowledge was poor and they had many misconceptions [15]. The situation among urban and other Kenyan male groups is likely, however, to be different. An in-depth analysis of DHS data from 58 men’s surveys across 18 countries in Africa, Asia, Latin America and the Caribbean further highlights the varied knowledge on contraceptive methods by age, marital status and educational level [45]. It is important to note that there are only two modern contraceptive methods for men which are the male condom and vasectomy, which registered at 3.1 and 0.0%, respectively, among all sexually active women respondents from the 2014 KDHS [1]. All the same, efforts are being made to increase the uptake of vasectomy with Kenya being among the 40 countries worldwide that will be commemorating World Vasectomy Day on November 18^th^ 2016 [46].

Men should be considered not only as women’s partners, but also as individuals with distinct reproductive histories and desires of their own [24]. Adopting a similar methodology to the Uganda study [32], this paper seeks to understand the determinants of modern contraceptive use among sexually active men, by exploring factors (explanatory variables) that are correlated with modern contraceptive use (outcome variable). Further, it will explore the relative strength of these associations in bivariate and multivariate models. Findings from this study will be of significant importance not only to the Government of Kenya, but also to partner organizations working on family planning in Kenya to inform programs that influence contraceptive use decisions among men and women. Additionally, the paper seeks to contribute to the discussion of men’s place in reproductive health research.

In order to inform our analysis, we used a customized conceptual framework to understand the determinants of modern contraceptive use among men, this builds on existing knowledge on factors associated with contraceptive use. We hypothesize that factors associated with modern contraceptive use operate at different levels.
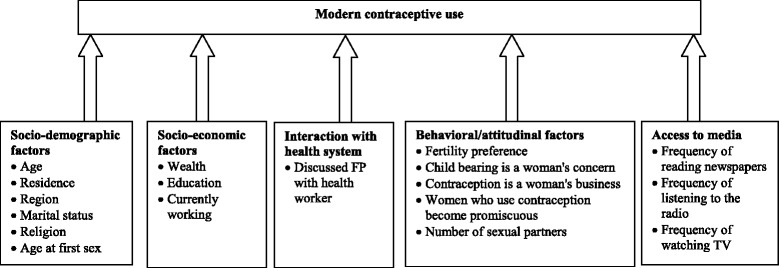


In our framework we consider the socio-demographic factors, socio-economic factors, interaction with the health system, access to media, and behavioural/attitudinal factors as the main potential influencers of modern contraceptive use among men. The socio-demographic factors are hypothesized to operate directly to influence modern contraceptive use, and so do socio-economic factors, interaction with the health system, behavioral/attitudinal factors and the factors related to access to media. We therefore fit five models to explore these relationships in multinomial models.

## Methods

### Source of data

The data source is the nationally representative 2014 Kenya Demographic and Health Survey (DHS) of men aged 15–54 years. The survey was designed to provide population and health indicator estimates at the national, provincial level and county level. The Kenya DHS applied probability sampling to provide nationally representative samples of men aged 15–54 years. The survey was conducted by the Kenya National Bureau of Statistics and ICF International. Interviews with men covered 12,819 of the eligible 14,217 men, yielding a response rate of 90.2%. Data was weighted in order to adjust for differences in probability of selection and to adjust for non-response. As of April 2016, this was the latest survey data available for Kenya. This analysis is restricted to the 9,514 (weighted) men who reported being sexually active in the 12 months prior to the survey, as they were likely to report either doing something or not to avoid or delay a pregnancy. We excluded from analysis men who reported that either them or their partners were infecund or sterile as they were not exposed to the risk of pregnancy.

### Study variables

The men’s questionnaire reports contraceptive use among men through the following question, ‘Are you currently doing something or using any method with any partner to delay or avoid a pregnancy?’ those who responded with a ‘*yes’* were further asked to state the method they were personally using or their partner(s) were using. The options listed included: not using, pill, IUD, injections, condom, female sterilization, male sterilization, implants/norplant, lactational amenorrhea, periodic abstinence, female condom, and withdrawal. Out of these categories, the outcome variable, modern contraceptive use, was coded as a three outcome variable as: ‘*traditional/no method*’ for those who reported current non-use of modern contraceptive methods or use of traditional or natural methods (such as periodic abstinence, lactational amenorrhea and withdrawal) which are not effective in pregnancy prevention; ‘*partner method*’ for those who reported using a method through their partner (such methods include pill, IUD, injections, female sterilization and norplant); and ‘*male method*’, for those who reported using male only methods (such as condoms and male sterilization).

The explanatory variables were grouped into categories hypothesized to influence modern contraceptive use, as shown in the conceptual framework above. The socio-demographic factors (age, residence, region, marital status, religion and age at first sex); socio-economic factors (wealth; education and employment status); interaction with health system (discussed FP with health worker); behavioural/attitudinal factors (fertility preference, child bearing is a woman’s concern, contraception is a woman’s business, women who use contraception become promiscuous and number of sexual partners); and access to media (frequency of reading newspapers, frequency of listening to the radio and frequency of watching TV) are hypothesized to influence modern contraceptive use as shown in the conceptual framework above.

### Data analysis

Data analysis was carried out using STATA v.14, descriptive statistics were used to provide sample characteristics including socio-demographic characteristics, exposure to family planning messages, interaction with health system and sexual behaviour. Secondly, we carried out bivariate analysis between each explanatory variable and the outcome variable to determine the variables to include in the five multivariate models as informed by our conceptual framework. Explanatory variables that were significantly associated with the outcome variable at 5% level of significance or less, were included in the multinomial logistic regression models to further assess variables that were statistically associated with modern contraceptive use. Bivariate analysis was used to assess the individual relationship of each explanatory variable with modern contraceptive use while multivariate analysis was used to assess relationships while controlling for other explanatory variables. The outcome variable, a three outcome variable coded as none/traditional method, partner method and male method was fitted in multinomial models to predict the determinants of modern contraceptive use among sexually active men. In total five models were fitted as informed by our conceptual framework, Model I assessed the determinants of contraceptive use in relation to socio-demographic factors, Model II controlled for the effects of socio-economic factors, Model III controlled for interaction with the health care system. Model IV assessed the effects of behavioural/attitudinal factors and Model V, controlled for media access factors. All analyses were weighted to account for differences in sampling probabilities.

## Results

### Sample description

Table 1 shows the description of 9,514 sexually active men aged 15–54 years from Kenya who participated in the 2014 Kenya DHS. Thirty-nine percent (39%), reported using no method or a traditional method while about 36% and 25% of the respondents reported currently using a partner and male method, respectively. Slightly more than half (54%) of the respondents resided in rural areas and most (63%) of the men were currently married. Nearly all the respondents had attended school, with 48% having attained primary education whereas 49% had secondary or higher education. A large proportion of men (91%) were engaged in some income generating activity in the 12 months prior to the survey. About 39% had at least three children and about a third (31%) desired to have another child in future while 37% reported having no regular partners. Responses on attitudinal statements on gender norms showed that a vast majority of the men disagreed with the statements, ‘contraception is a woman’s business’ (86%), and ‘women who use contraception become promiscuous’ (68%). However, only a small proportion (13%) had discussed family planning with a health worker. The full characteristics of respondents are shown in Table 1.

**Table 1:** Sample characteristics of sexually active men 15–54 years in Kenya [Weighted]

Characteristics	Percent (%)	N
Contraceptive use		
None/traditional	38.6	3,673
Partner method	36.4	3,462
Male method	25.0	2,378
Socio-demographic factors		
Age		
15–24	24.8	2,358
25–34	34.7	3,297
35–54	40.6	3,858
Living children		
None	31.0	2,951
1-2	30.2	2,875
3+	38.8	3,688
Age of last child		
No child	31.2	2,965
0–2 years	33.1	3,147
3+ years	35.7	3,401
Residence		
Urban	46.1	4,382
Rural	53.9	5,132
Region		
Nairobi	10.0	951
Central	1.1	102
Coast	15.1	1,434
Eastern	13.5	1,289
Nyanza	25.7	2,444
Rift valley	8.9	847
Western	11.7	1,114
North Eastern	14.0	1,332
Marital status		
Never married	30.5	2,900
Currently married	63.2	6,012
Formerly married	6.3	601
Religion		
Catholic	22.8	2,170
Potestant	67.0	6,379
Muslim	5.4	513
No religion	4.7	451
Number of wives		
No wives/partners	36.8	3,501
1 wife	59.5	5,663
2 and more wives	3.7	349
Age at first marriage		
Never married	30.5	2,900
11–24 years	40.3	3,833
25 and more years	29.2	2,780
Age at first intercourse		
Less than 14 years	15.2	1,447
14–17 years	45.3	4,313
18–24 years	34.8	3,310
25 and more years	4.7	443
Marital duration		
Never married	30.5	2,900
0–9 years	29.9	2,844
10+ years	39.6	3,769
Socio-economic factors		
Wealth index		
Poor	23.1	2,195
Medium	30.9	2,943
Rich	46.0	4,376
Education		
None	3.1	295
Primary	47.7	4,541
Secondary/Higher	49.2	4,678
Currently working		
No	9.4	896
Yes	90.6	8,617
Interaction with health system		
Discussed FP with health worker		
No	87.2	8,297
Yes	12.8	1,216
Behavioural/attitudinal factors		
Fertility preference		
Want another child	31.3	2,977
Undecided	2.0	186
Want no more	29.4	2,800
No regular partner	37.3	3,551
Contraception is a woman’s business		
Disagree	85.9	8,173
Agree	12.9	1,227
Don’t know	1.2	114
Women who use contraception become promiscous		
Disagree	66.7	6,342
Agree	29.4	2,800
Don’t know	3.9	372
Number of sexual partners		
1 partner	9.8	929
2-5 partners	54.6	5,197
6+ partners	35.6	3,388
Access to media		
Frequency of reading newspaper/magazine		
Not at all	35.2	3,352
Less than once a week	21.2	2,015
At least once a week	43.6	4,147
Frequency of listening to radio		
Not at all	5.0	480
Less than once a week	7.6	721
At least once a week	87.4	8,312
Frequency of watching TV		
Not at all	21.1	2,009
Less than once a week	17.0	1,613
At least once a week	61.9	5,892
Total (N)	100.0	9,514

### Prevalence of modern contraceptive use among sexually active men

Table 2 shows the prevalence of modern contraceptive use in relation to selected factors categorized as socio-demographic status, socio-economic status, interaction with health care system, attitudinal/behavioural and media access of sexually active men. Considering the socio-demographic characteristics, there exists a significant positive association between age and use of partner methods. Men over 25 years were over three times as likely to use a partner method as compared to using traditional/no method. Conversely, these men were less likely to use a male method than traditional/no method, compared to younger men aged 15–24. Men with at least one child were more likely to report use of a partner method, but were less likely to report using a male method compared to those who had no children. Men whose last child was aged at least three years were five times more likely to report using a partner method than traditional/no method. Among these men with older children, reported use of a male method was much lower as compared to using traditional/no method. There was a significant difference between urban and rural men, where rural men were less likely to report use of a partner method compared to using traditional/no method, likewise, these men were less likely to report use of a male method compared to using traditional/no method than their urban counterparts. Contraceptive use varied by region of residence where men from Central were less likely to report a partner method as opposed to using traditional/no method. Men from Coast, Eastern, Rift Valley, Western and North Eastern were all more likely to report use of a partner method than a traditional/no method compared to those from Nairobi. Regarding marital status, currently married men were four times more likely to report a partner method than traditional/no method but less likely to use a male method. Men in monogamous marriages, and those in marriage for less than a year and onwards, were more likely to report using a partner method than using traditional/no method.

**Table 2:** Bivariate association between modern contraceptive use and various background characteritics

	Partner method vs. None/traditional method			Male method vs. None/traditional method		
Characteristics						
Socio-demographic factors						
Age						
15–24	1.00			1.00		
25–34	3.83	***	[3.11–4.72]	0.41	***	[0.35-0.47]
35–54	3.57	***	[2.90-4.39]	0.14	***	[0.12-0.17]
Living children						
None	1.00			1.00		
1-2	5.58	***	[4.29-7.27]	0.27	***	[0.22-0.33]
3+	4.47	***	[3.54-5.66]	0.11	***	[0.09-0.14]
Age of last child						
No child	1.00			1.00		
0–2 years	4.68	***	[3.64-6.03]	0.15	***	[0.12-0.19]
3+ years	5.26	***	[4.17-6.63]	0.20	***	[0.16-0.24]
Residence						
Urban	1.00			1.00		
Rural	0.71	***	[0.61-0.84]	0.81	*	[0.67-0.97]
Region						
Nairobi	1.00			1.00		
Central	0.09	***	[0.04-0.19]	0.08	***	[0.04-0.19]
Coast	1.74	***	[1.32-2.29]	1.44	*	[1.09-1.90]
Eastern	2.30	***	[1.75-3.03]	1.61	***	[1.21-2.14]
Nyanza	1.23		[0.98-1.54]	1.28	*	[1.01-1.63]
Rift valley	1.59	**	[1.17-2.18]	1.24		[0.94-1.64]
Western	1.85	***	[1.45-2.36]	1.90	***	[1.47-2.46]
North Eastern	2.35	***	[1.67-3.31]	2.08	**	[1.32-3.29]
Marital status						
Never married	1.00			1.00		
Currently married	4.37	***	[3.51-5.45]	0.09	***	[0.07-0.10]
Formerly married	1.90	***	[1.29-2.79]	0.53	***	[0.40-0.69]
Religion						
Catholic	1.00			1.00		
Potestant	1.03		[0.87-1.22]	0.91		[0.77-1.08]
Muslim	0.33	***	[0.24-0.45]	0.46	**	[0.32-0.66]
No religion	0.48	***	[0.34-0.69]	0.72		[0.51-1.03]
Number of wives						
No wives/partners	1.00			1.00		
1 wife	3.77	***	[3.12-4.57]	0.09	***	[0.08-0.11]
2 and more wives	2.59	***	[1.81-3.70]	0.10	***	[0.06-0.16]
Age at first marriage						
Never married	1.00			1.00		
11–24 years	4.10	***	[3.26-5.16]	0.14	***	[0.11-0.16]
25 and more years	4.24	***	[3.35-5.38]	0.11	***	[0.09-0.14]
Age at first intercourse						
Less than 14 years	1.00			1.00		
14–17 years	0.97		[0.78-1.20]	0.86		[0.70-1.07]
18–24 years	1.16		[0.94-1.45]	0.83		[0.67-1.04]
25 and more years	0.78		[0.57-1.07]	0.27	***	[0.16-0.44]
Marital duration						
Never married	1.00			1.00		
0–9 years	4.31	***	[3.39-5.48]	0.14	***	[0.11-0.17]
10+ years	4.05	***	[3.23-5.09]	0.11	***	[0.09-0.14]
Socio-economic factors						
Wealth index						
Low	1.00			1.00		
Medium	2.19	***	[1.87-2.58]	1.71	***	[1.44-2.03]
High	2.67	***	[2.25-3.18]	1.81	***	[1.48-2.22]
Education						
None	1.00			1.00		
Primary	6.63	***	[4.25-10.33]	4.15	***	[2.61-6.60]
Secondary/Higher	9.27	***	[5.90-14.57]	7.04	***	[4.38-11.31]
Currently working						
No	1.00			1.00		
Yes	4.55	***	[3.23-6.40]	0.50	***	[0.41-0.62]
Interaction with health system						
Discussed FP with health worker						
No	1.00			1.00		
Yes	1.71	***	[1.42-2.07]	0.84		[0.65-1.09]
Behavioral/attitudinal factors						
Fertility preference						
Want another child	1.00			1.00		
Undecided	1.30		[0.82-2.05]	1.25		[0.66-2.38]
Want no more	1.36	***	[1.16-1.60]	1.13		[0.83-1.54]
No regular partner	0.36	***	[0.29-0.44]	11.21	***	[8.63-14.57]
Contraception is a woman’s business						
Disagree	1.00			1.00		
Agree	0.74	**	[0.61-0.89]	0.81	*	[0.66-0.99]
Don’t know	0.09	***	[0.03-0.28]	0.50	*	[0.29-0.85]
Women who use contraception become promiscous						
Disagree	1.00			1.00		
Agree	0.60	***	[0.52-0.69]	1.01		[0.86-1.18]
Don’t know	0.42	***	[0.30-0.58]	0.68	*	[0.48-0.97]
Number of sexual partners						
1 partner	1.00			1.00		
2–5 partners	2.02	***	[1.61-2.53]	1.19		[0.96-1.47]
6+ partners	2.37	***	[1.87-3.01]	1.11		[0.87-1.40]
Access						
Frequency of reading newspaper/magazine						
Not at all	1.00			1.00		
Less than once a week	1.49	***	[1.23-1.81]	1.38	**	[1.11-1.71]
At least once a week	2.11	***	[1.83-2.45]	1.56	***	[1.31-1.87]
Frequency of listening to radio						
Not at all	1.00			1.00		
Less than once a week	1.62	*	[1.01-2.60]	2.04	***	[1.37-3.04]
At least once a week	2.56	***	[1.80-3.63]	2.32	***	[1.66-3.23]
Frequency of watching TV						
Not at all	1.00			1.00		
Less than once a week	1.65	***	[1.35-2.01]	1.65	***	[1.31-2.09]
At least once a week	2.29	***	[1.92-2.73]	1.95	***	[1.61-2.35]

^*^ *p* < .05; ** *p* < .01; ***p < .001

Associations with socio-economic factors show that men with higher levels of education and wealth were more likely to use a partner method as compared to using traditional/no method. The analysis further showed that interaction with the health care system (having discussed FP with a health worker) increased the likelihood of men to report using a partner method than use traditional/no method. Whereas behavioural/attitudinal factors show that men who did not desire more children were more likely to report a partner method. Use of partner method was less common among men who reported having no regular partners. However their reported use of a male method was over 11 times as compared to using traditional/no method. Partner method use was negatively associated with agreeing with the following attitudinal statements: contraception is a woman’s business, and women who use contraception become promiscuous. Men who read newspapers/magazines at least once week and those who watched TV at least once a week were more likely to report using a partner method as opposed to using traditional/no method.

### Determinants of modern contraceptive use

Multinomial regression shown on Table 3 was applied using five models to assess the effect of explanatory factors on modern contraceptive use among sexually active men. Model I controls for the effect of socio-demographic factors and shows that men aged 25 years and above were more likely to report use of a partner method than use of a traditional/no method compared to those under 25 years. Men from Central were 0.9 times and 0.8 times, *p* < 0.001 less likely to report use of partner method and male method respectively than use traditional/no method compared to those from Nairobi. On the other hand, men from Coast (1.7 times, *p* < 0.001), Eastern (2 times, *p* < 0.001) and North Eastern (1.7 times, *p* < 0.05), were more likely to report use of a partner method than use traditional/no method compared to men from Nairobi. Moreover, men from Coast, Eastern, Rift Valley and North Eastern were more likely to use a male method than using traditional/no method. Currently married and formerly married men were more likely to report using a partner method but less likely to use a male method than using traditional/no method. Muslim men and men reporting no religion were less likely to report use of partner method as opposed to using traditional/no method than those of the Catholic faith.

**Table 3:** Odds ratio of modern contraceptive use among sexually active men in Kenya

	Partner method vs. None/traditional method		Male method vs. None/traditional method				Partner method vs. None/traditional method			Male method vs. None/traditional method			Partner method vs. None/traditional method			Male method vs. None/traditional method		Partner method vs. None/traditional method			Male method vs. None/traditional method			Partner method vs. None/traditional method			Male method vs. None/traditional method		
Characteristics																													
Socio-demographic factors	Model I						Model II						Model III					Model IV						Model V					
Age																													
15–24	1.00			1.00																									
25–34	2.07	***	[1.60-2.68]	1.08		[0.90-1.30]																							
35–54	1.66	***	[1.27-2.18]	0.80		[0.61-1.04]																							
Residence																													
Urban	1.00			1.00																									
Rural	0.75	***	[0.64-0.88]	0.91		[0.76-1.09]																							
Region																													
Nairobi	1.00			1.00																									
Central	0.12	***	[0.05-0.26]	0.18	***	[0.08-0.40]																							
Coast	1.65	***	[1.25-2.19]	1.40	*	[1.00-1.95]																							
Eastern	2.00	***	[1.47-2.72]	1.63	**	[1.18-2.26]																							
Nyanza	1.10		[0.86-1.42]	1.32		[0.98-1.77]																							
Rift valley	1.40		[1.00-1.96]	1.42	*	[1.01-2.00]																							
Western	1.47		[1.11-1.95]	2.63	***	[1.92-3.62]																							
North Eastern	1.71	*	[1.17-2.49]	1.98	***	[1.25-3.13]																							
Marital status																													
Never married	1.00			1.00																									
Currently married	3.38	***	[2.56-4.47]	0.09	***	[0.07-0.12]																							
Formerly married	1.48	*	[0.98-2.22]	0.61	***	[0.46-0.81]																							
Religion																													
Catholic	1.00			1.00																									
Potestant	1.07		[0.90-1.28]	0.93		[0.76-1.14]																							
Muslim	0.52	***	[0.36-0.77]	0.89		[0.60-1.32]																							
No religion	0.58	**	[0.41-0.82]	0.74		[0.50-1.10]																							
Age at first intercourse																													
Less than 14 years	1.00			1.00																									
14–17 years	0.94		[0.75-1.18]	0.97		[0.77-1.22]																							
18–24 years	1.03		[0.81-1.31]	1.13		[0.89-1.44]																							
25 and more years	0.68	*	[0.49-0.95]	0.76		[0.95-2.44]																							
Socio-economic factors																													
Wealth index																													
Low							1.00			1.00																			
Medium							1.85	***	[1.57-2.19]	1.48	***	[1.23-1.78]																	
High							2.10	***	[1.74-2.53]	1.39	**	[1.13-1.72]																	
Education																													
None							1.00			1.00																			
Primary							5.00	***	[3.18-7.85]	3.70	***	[2.30-5.94]																	
Secondary/Higher							6.19	***	[3.89-9.82]	5.57	***	[3.42-9.06]																	
Currently working																													
No							1.00			1.00																			
Yes							4.48	***	[3.16-6.35]	0.52	***	[0.42-0.64]																	
Interaction with health system																													
Discussed FP with health worker																													
No													1.00			1.00													
Yes													1.71	***	[1.41-2.07]	0.84	[0.65-1.09]												
Behavioral/attitudinal factors																													
Fertility preference																													
Want another child																		1.00			1.00								
Undecided																		1.29		[0.81-2.05]	1.24		[0.65-2.37]						
Want no more																		1.31	***	[1.12-1.54]	1.10		[0.81-1.50]						
No regular partner																		0.39	***	[0.32-0.48]	11.87	***	[9.08-15.51]						
Contraception is a woman’s business																													
Disagree																		1.00			1.00								
Agree																		0.87		[0.71-1.06]	0.82		[0.65-1.04]						
Don’t know																		0.18	**	[0.05-0.65]	0.35	***	[0.19-0.64]						
Women who use contraception become promiscous																													
Disagree																		1.00			1.00								
Agree																		0.64	***	[0.55-0.74]	0.92		[0.76-1.10]						
Don’t know																		0.58	**	[0.41-0.83]	0.83		[0.54-1.27]						
Number of sexual partners																													
1partner																		1.00			1.00								
2–5 partners																		1.69	***	[1.34-2.14]	1.65	***	[1.28-2.12]						
6+ partners																		1.93	***	[1.51-2.48]	1.72	***	[1.32-2.24]						
Access																													
Frequency of reading newspaper/magazine																													
Not at all																								1.00			1.00		
Less than once a week																								1.29	*	[1.06-1.57]	1.19		[0.96-1.47]
At least once a week																								1.63	***	[1.39-1.90]	1.24	*	[1.02-1.49]
Frequency of listening to radio																													
Not at all																								1.00			1.00		
Less than once a week																								1.46		[0.91-2.36]	1.83	**	[1.23-2.73]
At least once a week																								1.82	***	[1.26-2.61]	1.77	***	[1.25-2.50]
Frequency of watching TV																													
Not at all																								1.00			1.00		
Less than once a week																								1.39	**	[1.12-1.71]	1.45	**	[1.14-1.84]
At least once a week																								1.67	***	[1.37-2.04]	1.66	***	[1.35-2.04]

^*^ *p* < .05; ** *p* < .01; ***p < .001

In Model II we controlled for socio-economic factors, men belonging to upper wealth quintile households were more likely to report using male and partner methods than traditional/no method compared to those from poor households. Men reporting at least primary education were more likely to report use of partner or male method than traditional/no method compared to those with no education. Men who were currently working were more likely to report use of a partner method (4.5 times, *p* < 0.001), but were less likely (0.5 times, *p* < 0.001) to report use of a male method than traditional/no method compared to those who were not working. Model III controlled for interaction with a health system. In this model, men who had a discussion with a health worker were more likely to report usage of a partner method (1.7 times, *p* < 0.001) as opposed to traditional/no method. In model IV, we controlled for behavioral/attitudinal factors related to fertility preference. Here, men who desired no more children were 1.3 times (*p* < 0.001) more likely to report use of a partner method as opposed to usage of traditional/no method. On the other hand, men who had no regular partners were less likely to report use of a partner method but were 12 times (*p* < 0.001) more likely to report use of a male method than traditional/no method compared to those who reported wanting another child. With respect to the number of sexual partners, men who reported more than one sexual partner were more likely to use partner or male method as opposed to traditional/no method. Similar to the bivariate model, partner method use was negatively associated with agreeing with attitudinal statements on gender norms. Lastly, Model V controlled for access to media. It is evident from this model that men who read a newspaper/magazine at least once a week were more likely to report either use of partner method (1.6 times, *p* < 0.001) or male method (1.2 times, *p* < 0.05) than using traditional/no method. Radio listenership of at least once a week increased the likelihood to use partner or male method (1.8 times, *p* < 0.001), similarly, watching television at least once a week increased the likelihood to use partner or male method (1.7 times, *p* < 0.001) compared to use of traditional/no method than those who never watched television at all.

## Discussion

This paper examined the correlates of using modern contraceptive methods among sexually active men in the reproductive bracket of 15–54 years using the 2014 Kenya DHS data. The findings from the bivariate logistic regression somewhat conform to the general literature on contraceptive use among women. In line with these studies, the bivariate analysis found that a number of socio-demographic and socio-economic factors are associated with contraceptive use (both partner and male methods) among sexually active men. These factors include age (men >25 years), number of children (at least three children), education and wealth status (at least primary education and high socio-economic status), marital status (currently married), marriage type (monogamous) gender norms (positive gender attitudes), place of residence (urban), region of residence (Central, Eastern and Coast), religion (being a Muslim) discussion with a health worker, listenership to radio, reading a newspaper and watching television. Like studies conducted among women in Kenya, men from rural areas were both less likely to use partner or male methods compared to their urban counterparts. Additionally, there were regional differentials with male residents from Central, Coast and Eastern provinces being more likely to use a partner method than men from North Eastern province. Although age, marital status, religion and place of residence were associated with contraceptive use in nearly all models, other factors specifically, education, wealth status, positive gender norms and access to media were found to be important predictors of contraceptive use.

Overall, one consistently significant factor associated with contraceptive use after controlling for different factors in the five models was being a Muslim (Northern Eastern province is predominantly Muslim). This finding echoes similar findings from the Kenya DHS which showed the influence of religion on contraceptive use. Equally, studies conducted among women in Northern Eastern region have documented disproportionately low contraceptive use with high fertility levels (5.9 children per woman) as compared with other provinces in Kenya [8]. Factors underlying high fertility rates are linked to poor socio-economic indicators, more so, religion (the region is predominantly Muslim) as well as adherence to various cultural practices which have been noted to undermine family planning programs in the region. Similarly, North Eastern province has well over three quarters of its population in the lowest quintile and educational attainment is low (49.2% for males and 69.0% for females have no edication) compared to other provinces in Kenya [1, 47].

Our analysis also found that a vast majority of men rely on partner contraceptive methods as opposed to male methods. As described above, and echoing past literature, the predictors of contraceptive use among males are to a large extent related to male educational level and higher socio-economic status which are also important determinants for contraceptive use among women. In our analysis, we found a close association between male method use and men’s wealth status, discussion with a health worker, number of partners as well as access to media. This potentially shows that in general the use of male methods increases with higher socio-economic status, perception of risk, less cultural conservatism and a favorable environment shown by the intervening variables [48].

Importantly, findings from this study draw attention to the role men may play as co-decision-makers relating to fertility and fertility control. For a long time, family planning programs and related research have until recently exclusively focused attention on women as the primary targets for information, education and communication for information and use. Men on the other hand, are viewed as having a marginal role to play on contraceptive practice. Consequently, the role of women who are perceived to be centrally placed in contraceptive practice has significantly increased. Despite this, men’s positive or negative attitudes potentially determines women’s decision making to use contraceptives [49–51]. In particular, gender attitudes are important in couples’ decisions about acceptance and use of contraceptives. Since men remain important decision makers in contraceptive use, they should be adequately involved in population issues to increase their understanding hence support for contraceptive use [52]. Studies on gender norms indicate that perceived spousal disapproval of contraceptive use was enough to increase unmet need for contraception. Further, lack of communication between couples about their reproductive intentions has been linked with higher unmet need for contraception [53]. Negative gender attitudes among men have been reported elsewhere to restrict women’s uptake of contraceptives, on the other hand, men with positive gender sensitive decision making skills were more likely to support contraceptive use [21]. In this study, some predictors of contraceptive use among sexually active men (such as communication with a health worker, number of living children, wealth status, education and marital status) have been observed in similar analysis of a male cohort in Uganda [32]. Health workers remain important in promoting contraceptive uptake by providing information that make couples make informed choices thereby resulting to contraceptive compliance [32]. Additionally, it has been reported that targeted communication by peer educators and health personnel have been positively associated with use of family planning among men [54].

In sum, findings from this study suggest that improving the socio-economic and demographic factors is essential. Family planning programs should at all times be inclusive and target both men and women in equal measure. Programs focusing on improving contraceptive use among men should consider utilizing multiple approaches to reach different segments of the male population. Integrating men in reproductive health issues will lead to greater uptake of contraceptives and also break the power dynamics and gender norms that discourage contraceptive uptake. DHS surveys can provide more information on the participation of men in reproductive health issues by including more topics around men’s health, access to care, social support and fertility desires. Like most cross-sectional surveys, the major limitation of this study in the ability to draw reliable measures of modern contraceptive use may be limited by the nature of information reported at the time of interview. It is therefore not possible to draw robust conclusions on the influence of various background factors on modern contraceptive use. However, despite this shortcoming, the paper provides an interesting contribution to the debate on the role of men in family planning, an area that has not been fully explored in the Kenyan context.

## Conclusions

The findings presented here moved beyond the traditional DHS analysis of modelling contraceptive use among women. We considered factors contributing to modern contraceptive use among sexually active men, namely the extent to which different explanatory variables affected the use of modern contraceptives. Men who had no education had a low degree of contraceptive awareness and were less likely to use modern contraceptives. This group of men seemed not to be properly informed about contraceptives as well as their benefits. Men from North-Eastern Kenya appeared to lag far behind other regions with uptake of modern contraceptives. Religion and gender attitudes also seem to shape behavior and practice on contraceptive use among men from North-Eastern Kenya. Our analyses suggest that interpersonal communication and mass media have a positive effect on modern contraceptive use. Provider–client interaction as well as dissemination of information through mass media could facilitate the dissemination of information and potentially increase knowledge and uptake of modern contraceptives. Similar efforts should focus on mass family planning sensitization campaigns targeting key sectors of the population where uptake of modern contraceptives remains low.

## Abbreviations

CPR: Contraceptive prevalence rate
DHS: Demographic and health survey
HIV/AIDS: Human immunodeficiency virus/Acquired immuno-deficiency syndrome
KDHS: Kenya demographic and health survey
TV: Television
